# Supplementary material for: Oral Fluids for the Early Detection of Classical Swine Fever in Commercial Level Pig Pens
Source: Viruses. 2024 Feb 20;16(3):318. doi: 10.3390/v16030318 (PMC10974009; doi:10.3390/v16030318)
Supplement: Supplementary file 1 [file viruses-16-00318-s001.zip › viruses-2854649-supplementary.pdf]

A

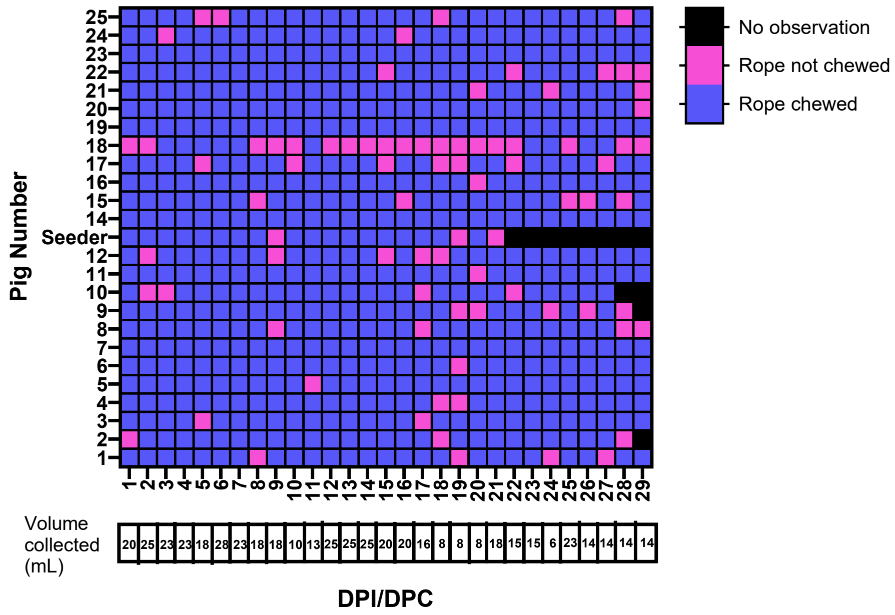

B

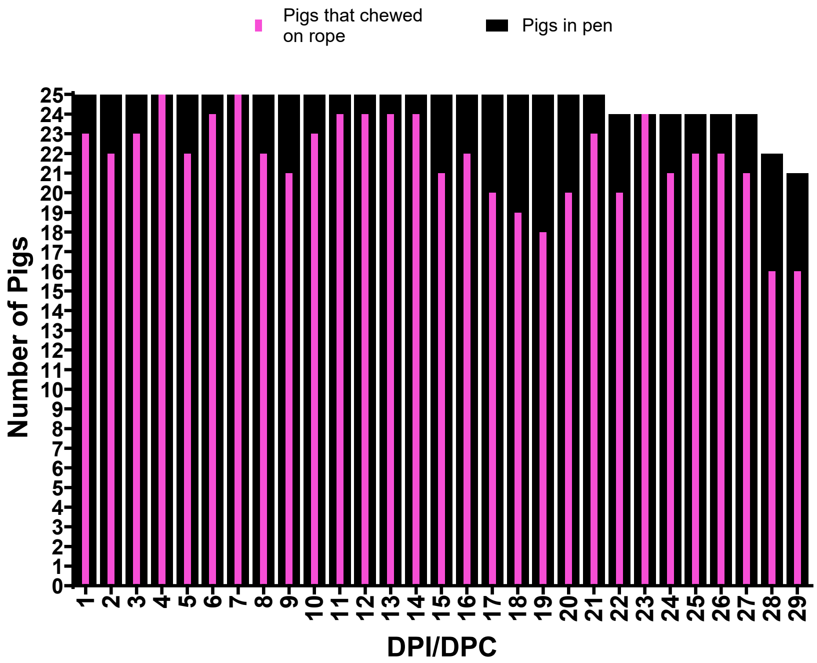

Supplementary Figure 1. Contribution of individual pigs to oral fluid collected in Experiment #1. (**A**) The heat map depicting pigs that chewed the ropes during 30 min collection time as observed through a CCTV camera. Also shown, the total volume of oral fluid collected each day. (**B**) The number of pigs that chewed on the rope compared to the total number of pigs in the pen on each day post-infection.

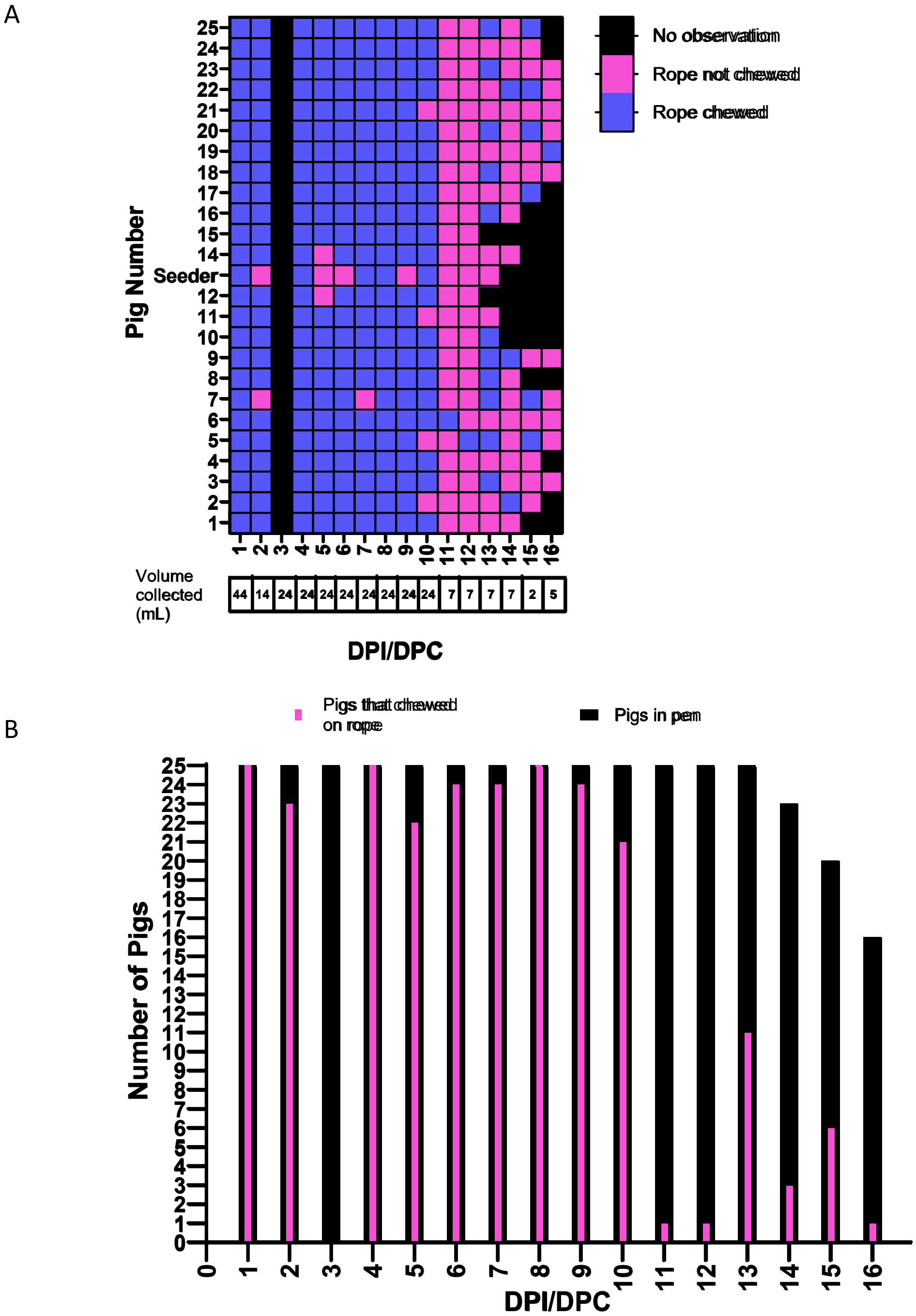

Supplementary Figure 2. Contribution of individual pigs to oral fluid collected in Experiment #2. (**A**) The heat map depicting pigs that chewed the ropes during 30 min collection time, as observed through a CCTV camera. On 3 dpi, no observations were conducted. Also shown, the total volume of oral fluid collected each day. (**B**) The number of pigs that chewed on the rope compared to the total number of pigs in the pen on each day post-infection.

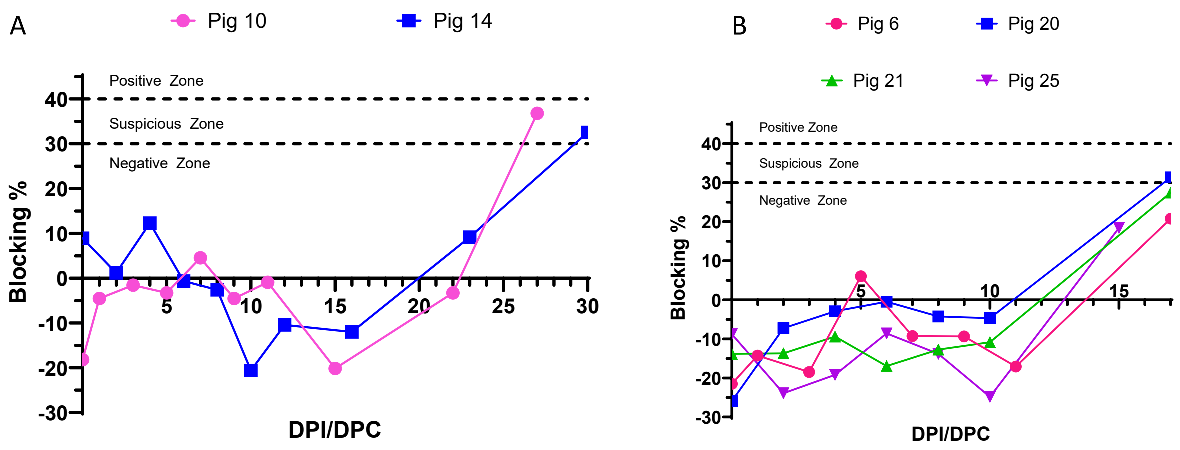

Supplementary Figure 3. The antibody response in pigs infected with (**A**) CSFV Pinillos and (**B**) Koslov. The cut-off values for the ELISA are indicated by dotted lines (<30%, negative; 30-40%, suspicious; ≥40% positive).
